# Case report: Treatment of a patient with STEMI and cardiogenic shock caused by RCA originating from LAD

**DOI:** 10.3389/fcvm.2022.1036274

**Published:** 2023-01-10

**Authors:** Qiang Niu, Yunhe Zhao, Haiying Li, Qilei Wang, Haijun Zhu, Chenglong Bi, Hui Zhang, Jian Wang, Cheng Cheng, Beibei Song, Chengwei Jin, Ming Lv, Bo Li

**Affiliations:** ^1^Department of Cardiology, Zibo Central Hospital, Zibo, China; ^2^Department of Medical, Zibo Central Hospital, Zibo, China

**Keywords:** AMI, percutaneous coronary intervention (PCI), anomaly, drug-coated balloon (DCB), ventricular fibrillation (VF)

## Abstract

The right coronary artery (RCA) originating from the left anterior descending artery (LAD) is a very rare variation of coronary artery anomaly. This kind of anomaly is usually considered to be clinically benign. Here, we present an acute myocardial infarction patient with a single coronary artery (SCA), in whom the LAD and RCA are both occlusive at the same time. He suffered from ventricular fibrillation, cardiogenic shock, and severe bradyarrhythmias many times. Fortunately, this patient survived from death through our effective medical procedures.

## Introduction

Coronary artery anomalies are present at birth, and they are relatively uncommon findings in coronary angiography. Rarely, only one coronary artery originates from the aortic trunk supplying the entire heart; as a rare variation, a single coronary artery (SCA) arises from the left sinus of Valsalva, and the right coronary artery (RCA) originates from the left anterior descending artery (LAD) (1, 2). This kind of anomaly is usually considered to be clinically benign (1). Here, we report the case of a patient with acute myocardial infarction and a SCA in whom the LAD and RCA were both occluded at the same time. This report is the first such an anomaly associated with a variety of serious complications. The patient underwent percutaneous coronary intervention two times to the LAD/RCA bifurcated lesion and finally completed revascularization half a year after symptom onset.

## Case report

**FIGURE 1:**
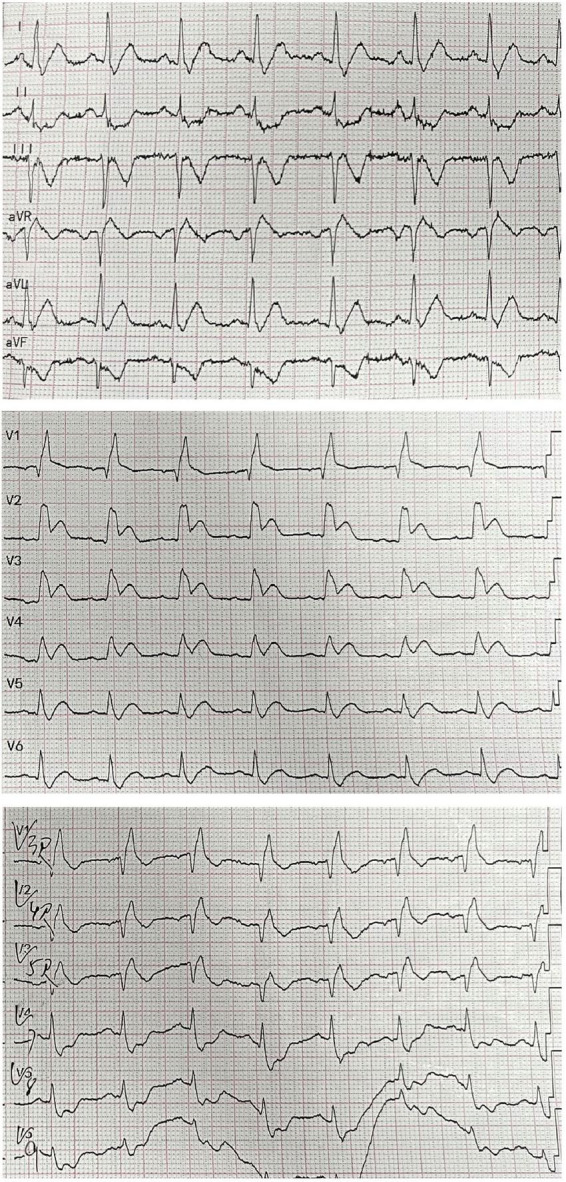
A 18-lead electrocardiogram of the patient showing ST elevation in V2–V4 leads with widespread inverted T-waves in inferior, posterior, and right ventricular leads.

**FIGURE 2:**
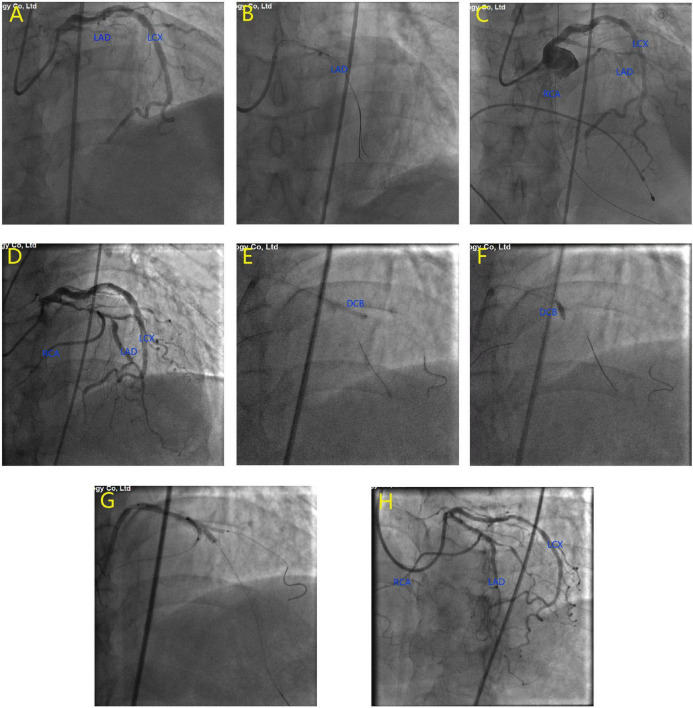
Emergency coronary angiography at the onset of acute myocardial infarction. The results showed that the proximal left anterior descending artery (LAD) was completely occluded **(A)**. Balloon dilatation was performed on the occlusive site **(B)**. TIMI grade 3 flow was restored in LAD and right coronary artery (RCA) **(C)**. The second coronary angiography at 4 months after myocardial infarction. The results showed high-grade lesion at the proximal LAD, as well as at LAD/RCA bifurcation and LAD/diagonal bifurcation **(D)**. Drug-eluting balloons were used to dilate the diagonal branch **(E)** and the RCA **(F)**. A drug-eluting stent in LAD and 2 balloons in D1 and RCA were dilated simultaneously for kissing balloon inflation **(G)**. Final imaging results after procedure **(H)**.

The patient was a 76-year-old man with a history of cerebral infarction. He was admitted to the emergency room because of sudden and severe chest pain over the previous 3 h. The first electrocardiography showed obvious ST elevation in V2–V4 (Figure 1), and his troponin I level was > 80 pg/ml, which exceeded the laboratory’s testing limit. The first blood pressure was 67/40 mmHg, and a high dose of dopamine and noradrenaline is used to maintain adequate blood pressure. Then, the patient suffered from sudden cardiac arrest and ventricular fibrillation several times, and chest compressions and electric defibrillation were performed urgently over the course of diagnosis and treatment. We decided to perform emergency coronary angiography. During coronary angiography, the left coronary ostium was located normally. Selective left coronary angiography demonstrated a normal left main coronary artery, ostial LAD occlusion, and 40% ostial left circumflex artery (LCX) lesions (Figure 2A). Multiple attempts to cannulate the right coronary ostium were unsuccessful. As the blood pressure could not be maintained merely depending on the use of vasopressor drugs, intra-aortic balloon pump (IABP) was performed to hold the blood pressure and prevent ischemia-reperfusion injury.

With the aid of IABP, a guide wire was used to cross the occluding lesion of the LAD, and TIMI grade 3 blood flow was achieved after balloon angioplasty. We found that the left circumflex is the dominant artery and RCA originated from the middle of the LAD (Figures 2B, C). As the ECG monitor showed a ventricular rate of 36 and iii° AV block occurred, we performed a temporary pacemaker. Due to massive thrombosis in the coronary arteries and unstable vital signs, we decided to stop the operation and initiate intensive antithrombotic therapy. After 2 weeks of therapy, the patient’s vital signs stabilized, his chest pain disappeared, and his heart failure improved. We gradually removed the IABP, temporary pacemaker, and vasopressors. Then, coronary artery bypass surgery (CABG) or re-percutaneous coronary intervention (PCI) was recommended for the patient, but he and his family rejected this recommendation.

**FIGURE 3:**
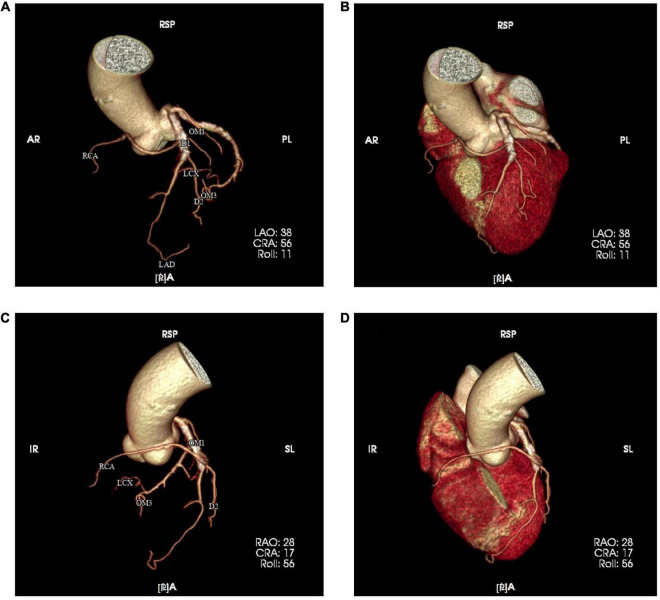
Coronary computed tomography angiography (CTA) was re-examined one month after PCI and resulting images **(A–D)** confirmed the anomalous origin of the right coronary artery.

Four months after discharge, the patient was advised again to undergo coronary angiography, and the results showed complex and severe triple bifurcated lesions, with 90% ostial LAD lesions, 80% ostial D1 lesions, and 70% ostial RCA lesions (Figure 2D). Following coronary angiography, we discussed the revascularization strategy. Two Sion blue wires were passed down the LAD, D1, and anomalous RCA, and two 2.5 × 20 mm drug-coated balloons were deployed in the D1 and RCA (Figures 2E, F). Then, the LAD/RCA/D1 bifurcation was treated with a 3.0 × 36 mm (10 atm) drug-eluting stent (DES) placed in the LAD and two Maverick 2.0 × 20 mm (6 atm) balloons placed in the D1 and RCA (Figure 2G), which were dilated simultaneously for kissing balloon inflation. Then, a proximal optimization technique (POT) was performed with a Quantum Maverick 3.0 × 15 mm high-pressure balloon placed in the LAD. With no symptoms of discomfort, PCI was completed, and the outcomes were satisfactory (Figure 2H). After 3 days, the patient was discharged home on aspirin, ticagrelor, and rosuvastatin. After a 1-month follow-up, we performed computed tomography angiography (CTA) of the coronary artery. Through CTA, we confirmed that the anomalous RCA originated from the middle of the LAD (Figure 3). It passed between the aorta and pulmonary artery in the group of L-II variants (1).

## Discussion

In previous studies, we learned that approximately 1.3% of the population has coronary artery anomalies (1), and these anomalies are found in 0.2 to 1.3% of patients undergoing coronary angiography and 0.3% of autopsy series (3). Coronary artery anomalies are considered to be the second most common cause of sudden cardiac death among young athletes. While an SCA, first defined by Hyrtl in (4), is a rare congenital anomaly, it occurs in approximately 0.024 to 0.066% of individuals (5). The first antemortem diagnosis was made by means of coronary angiography in 1967 (6).

To date, an increasing number of associated cases of an SCA have been reported, including an SCA arising from the LSV or RSV. Most of the published SCA case reports describe an RCA originating from the proximal or middle segment of the LAD, and this kind of anomaly was well-recognized as being clinically benign (1). Besides that, when the anomalous coronary artery courses between the aorta and pulmonary trunk, it is considered a malignant course and may cause sudden death (7). Here, we present a symptomatic case associated with the RCA originating from the LAD, and we did not find a similar case by searching PubMed. In such patients, LAD stenosis will also influence blood flow in the RCA. In the worst-case scenario, LAD stenosis can instantly cause simultaneous occlusion of both the LAD and RCA, resulting in a high risk of death and a poor prognosis. This 76-year-old man suffered from ventricular fibrillation, cardiogenic shock, and severe bradyarrhythmia many times throughout the diagnostic and treatment process; thus, this patient experienced all serious adverse events associated with LAD stenosis. Fortunately, this patient survived with the use of IABP, a temporary cardiac pacemaker, and an emergency operation. Many people survive such a complicated lesion. Selective revascularization was performed with DES and DCB 4 months later, and the patient was discharged from the hospital. We will continue to pay attention to the patient’s prognosis in the future.

Once a coronary artery anomaly is found in a patient, it is recommended that early preventive or even early treatment measures must be taken if there are lesions with a common origin, as seen in this patient. As the common origin is equivalent to LM, in the case of occlusion, the consequences would be severe and possibly fatal.

## Data availability statement

The original contributions presented in this study are included in the article/supplementary material, further inquiries can be directed to the corresponding authors.

## Ethics statement

Written informed consent was obtained from the individual(s) for the publication of any potentially identifiable images or data included in this article.

## Author contributions

All authors listed have made a substantial, direct, and intellectual contribution to the work, and approved it for publication.
